# In the Crosshairs: Investigating Lytic Granules by High-Resolution Microscopy and Electrophysiology

**DOI:** 10.3389/fimmu.2013.00411

**Published:** 2013-11-27

**Authors:** Varsha Pattu, Mahantappa Halimani, Min Ming, Claudia Schirra, Ulrike Hahn, Hawraa Bzeih, Hsin-Fang Chang, Lisa Weins, Elmar Krause, Jens Rettig

**Affiliations:** ^1^Institute of Physiology, Saarland University, Homburg, Saar, Germany

**Keywords:** cytotoxic T cells, lytic granules, TIRFM, structured illumination microscopy, correlative light and electron microscopy, SNARE proteins

## Abstract

Cytotoxic T lymphocytes (CTLs) form an integral part of the adaptive immune system. Their main function is to eliminate bacteria- and virus-infected target cells by releasing perforin and granzymes (the lethal hit) contained within lytic granules (LGs), at the CTL-target-cell interface [the immunological synapse (IS)]. The formation of the IS as well as the final events at the IS leading to target-cell death are both highly complex and dynamic processes. In this review we highlight and discuss three high-resolution techniques that have proven invaluable in the effort to decipher key features of the mechanism of CTL effector function and in particular lytic granule maturation and fusion. Correlative light and electron microscopy allows the correlation between organelle morphology and localization of particular proteins, while total internal reflection fluorescence microscopy (TIRFM) enables the study of lytic granule dynamics at the IS in real time. The combination of TIRFM with patch-clamp membrane capacitance measurements finally provides a tool to quantify the size of fusing LGs at the IS.

## Introduction

Cytotoxic T lymphocytes (CTLs) are a part of our body’s defense system. They eliminate bacterially and virally infected cells after coming in close contact with them to form an immunological synapse (IS). An IS is initiated when the antigen presented by MHC class 1 molecules on the infected cell forms a complex with the T cell receptors (TCR) on the CTL membrane. At least three antigenic peptide-MHC (p-MHC) complexes are needed to initiate the signaling cascade in CTLs that result in target-cell death (1). So, following p-MHC-induced TCR triggering, several morphological and functional changes occur within the CTL. Multiple receptor types and adhesion proteins aggregate at the contact zone to form supramolecular activation clusters [SMACs; (2)]. A central SMAC, composed of TCRs, Lck, ZAP70, and a host of other signaling molecules, is surrounded by a peripheral SMAC (pSMAC) that contains of adhesion molecules such as LFA1 and Talin, which are essential for stabilizing the contact between the CTL and the target-cell. The pSMAC is supported by an F-actin ring, formed after polar local actin polymerization and microfilament reorganization. The actin cytoskeleton supports the translocation of the microtubule organizing center (MTOC) toward the IS. The cascade of signaling events induced at the IS includes local and global calcium influx and ultimately leads to the MTOC-aided polarization of the lytic granules (LGs) to the IS (3).

Lytic granules are specialized lysosomes that contain cytotoxic molecules such as perforin and granzymes that induce target-cell death. They undergo a complex and still ill-defined set of maturation stages (see below) and are uniquely organized such that they are protected from the cytotoxic effects of the molecules they contain. Their acidic pH renders the pore-forming molecule perforin inactive. Following fusion at the IS, perforin is exposed to a neutral pH which results in its activation. Perforin is then bound to the target-cell membrane via its C2 lipid-binding domain and generates pores after oligomerization (4). Perforin also binds to the membrane of the CTL, but is cleaved by cathepsin B, which is incorporated into the CTL membrane when LGs fuse at the IS (5). Thus, perforin is only active in the membrane of the target-cell. CTLs are also protected from granzyme B (a serine protease) mediated killing by the expression of serpins (serine protease inhibitors) on their plasma membrane (6).

Lytic granules are the principal effectors of killer-cell function. Therefore, understanding the mechanism of their action and regulation is of great interest. Following polarization to the IS, LGs dock, prime, and finally fuse to release the perforin and granzymes at the IS. The docking, priming, and fusion steps of LGs at the IS are all tightly regulated and mediated by specific SNARE and SNARE-associated proteins (7). Several molecules essential for lytic granule exocytosis have been identified. Mutations within the genes of Rab27a, hMunc13-4, Syntaxin11, and Munc18-2 lead to defective CTL and NK cell function resulting in often lethal immune disorders: Griscelli syndrome (Rab27a) and FHL type-3, -4, and -5 (hMunc13-4, Syntaxin11, and Munc18-2, respectively). In the case of Rab27a and Munc13-4, in-depth studies using confocal imaging and high-resolution electron microscopy (EM) identified their precise function in LG fusion (8, 9). In addition, new insight on LG maturation was gained by elucidating the requirement of Munc13-4 for this process (10). Munc13-4 is an effector of Rab27a (11), and later studies using high-resolution total internal reflection fluorescence microscopy (TIRFM) showed that the Munc13-4 Rab27 complex is required for tethering LG at the plasma membrane (12). Detailed EM revealed that in patients suffering from Chediak–Higashi Syndrome (CHS), only lysosomes and not late multi-vesicular endosomes are enlarged (13). CHS is an autosomal recessive disease that results in defective T- and NK cell cytotoxicity, due to mutations in the *lyst* gene encoding for LYST protein. The accurate analysis from the EM studies in combination with confocal immunofluorescence imaging provided an elegant demonstration of the function of LYST and the molecular mishap behind the disease. Similarly, to investigate the precise function of Synaxin11 and Munc18-2 in CTLs, the molecular mechanism behind FHL-4 and 5 and to determine if Syntaxin11 is indeed the t-SNARE for the fusion of LG at the IS as has been hypothesized in several reports, TIRFM and EM would be the ideal methods of choice. Therefore, microscopic methods with high-resolution are essential in order to understand these spatially and temporally restricted processes at the IS. Furthermore, highly specific marker proteins for the different organelles involved, in particular LGs, are needed.

In this review we highlight a toolbox of techniques and molecules that should enable the quantitative analysis of LG biogenesis and fusion in CTLs.

## Investigating Granule Maturation, Its Types and Content through Electron Microscopy and Correlative Light and Electron Microscopy

**Figure 1:**
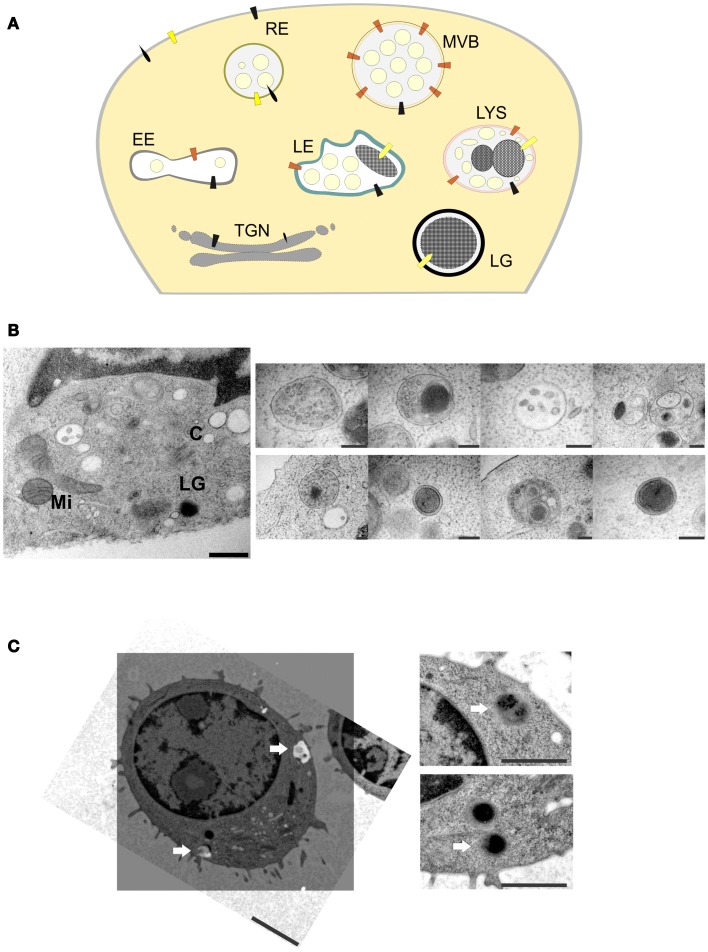
**(A)** Model of LG biogenesis in CTLs. RE, recycling endosomes; EE, early endosomes; TGN, trans-Golgi network; LG, lytic granule; LE, late endosomes; LYS, lysosomes; MVB, multi-vesicular bodies. **(B)** Left, ultrastructure of an immunological synapse of a mouse CTL formed after contact with anti-CD3/CD28 coated sapphire, mimicking the target-cell (scale bar: 500 nm). Right, EM micrographs of different organelles of unknown nature present at an immunological synapse (scale bar: 200 nm). LG, lytic granule; C, centriole; Mi, mitochondria; N, nucleus. **(C)** Representative correlative fluorescence electron microscopy (CLEM) image of a primary mouse CTL obtained from synaptobrevin2-mRFP knock in mice (19). Left, EM micrograph (ultrathin section of 80 nm) of a mouse CTL with the corresponding processed SIM-image. SIM-image was taken with a 63× Plan-Apochromat N. A. 1.52 with excitation light of 561 nm wavelength, z-stack of 0.2 μm step size were used to scan a 500 nm thick section. Arrows indicate synaptobrevin2-positive lytic granules (scale bar: 2 μm). Right, enlarged areas with the corresponding organelles (scale bar: 1 μm).

Only fully mature LGs fuse at the IS, but surprisingly little is known about the biogenesis of these LGs. Mature LGs contain many proteins, for example CD63 and the lysosomal-associated membrane proteins LAMP1, LAMP2, and LAMP3, that are also found on lysosomes (14, 15, 16). Therefore, they are also called secretory lysosomes (17) or lysosome-related organelles [LRO; (18)]. However, it remains unclear whether LGs are derived from lysosomes or whether they share a common precursor from which the two organelles mature independently (Figure 1A). Since they are only synthesized upon activation of the CTL, the presence of the lytic components perforin and granzymes seems to be a reliable indicator for the identification of mature LGs and their precursors. EM of cryosections revealed that perforin and granzymes are always colocalized in a homogenous population of LGs in mouse CTLs (15). As expected for the regulated secretory pathway, traces of the proteins can be found in the rough endoplasmic reticulum and in the trans-Golgi network (TGN), but not in endosomal compartments containing the mannose-6-phosphate receptor. These data indicate that at least the dense-core of LGs is derived directly from the TGN with no involvement of endosomal compartments. Interestingly, while in human CTLs the vast majority of perforin immunostaining was found in the dense-core of LGs, in mouse CTLs both perforin and granzyme B were preferentially detected in small internal vesicles surrounding the dense-core. It is currently unknown whether these small internal vesicles in LGs originate from fusion of immature LGs with late endosomes and/or multi-vesicular bodies (10, 18) or whether these vesicles fuse with the dense-core to add more lytic components. As shown in Figure 1B, high pressure freezing EM yields excellent preservation of intracellular organelles, but also reveals many different organelles which resemble LGs. Therefore, it is impossible to follow the maturation of LGs to the fully mature, fusogenic LGs from EM alone.

Immunogold EM has been the method of choice to verify the localization of proteins on structures such as LGs. However, it suffers from two serious drawbacks. First, it cannot be used in conjunction with osmium tetroxide, resulting in a very poor contrast of the images. Second, it relies on the availability of highly specific antibodies, which, for the majority of proteins, are still lacking. A recently developed alternative is correlative light and EM [CLEM; (20)]. Although it can be used with classical antibody-based immunostaining as well, its major advantage is that it allows the use of proteins tagged with green fluorescent protein (GFP) or derivatives for localization studies. These proteins can either be introduced into CTLs by overexpression or by the genetic replacement of the endogenous protein by a fusion protein in transgenic mice (knock in). An example for the latter strategy is shown in Figure 1C. Synaptobrevin2 is a vesicular SNARE protein that mediates the fusion of LGs with the plasma membrane at the IS (19). We genetically replaced the endogenous synaptobrevin2 gene by a synaptobrevin2-mRFP (monomeric red fluorescent protein) fusion protein. As a result, the purified primary CTLs from this knock in mouse contain red LGs. Importantly, since synaptobrevin2 is essential for the final step of LG function the red LGs from this mouse represent by definition fully mature LGs. It is interesting to note that although vesicles might look indistinguishable in electron micrographs, only a fraction of them are fully mature, i.e., fusogenic, as indicated by the red fluorescence (Figure 1C, lower right panel). Thus, it appears that CLEM is an excellent choice to identify the maturation steps and the associated morphologies of LGs.

Apart from LG biogenesis and maturation, there are still several open questions relating to some of the key components of LGs such as perforin. At least 50 different mutations in perforin are linked to familial hemophagocytic lymphohistiocytosis type-2 (FHL-2), emphasizing the importance of this protein in CTLs and NK cells. Perforin is synthesized as a 65 kDa precursor in the ER. Both the N-terminus and the C-terminus of perforin are distinct in their functional contributions. The N-terminus contains the signal peptide and the amino-terminal membrane attack complex perforin-like (MACPF)/cholesterol dependent cytolysin (CDC) domain. The C-terminal contains the C2-domain needed to bind membranes in a calcium-dependent manner. X-ray crystal structure of monomeric murine perforin and cryo-EM reconstruction of the entire perforin pore revealed new insights and flexibility into the mechanism of pore formation (21). This study showed, using EM, that the perforin MACPF domain in the pore is inside-out relative to the subunit arrangement in CDCs, thus raising new possibilities in the mechanism of perforin pore formation. The extreme C-terminus of perforin is needed for the transport of the protein from the ER to the Golgi. However, a mechanism of perforin sorting from the TGN has been postulated (22) and could be resolved using EM.

## Visualizing and Quantifying Lytic Granules at the IS by TIRFM

After having matured, the LG has to fulfill its central function to release its cytotoxic content into the synaptic cleft. To this end, LGs dock, prime, and then fuse (exocytose) with the plasma membrane. TIRFM has been widely used to study exocytic and endocytic events at the plasma membrane (23). TIRFM is an optical technique based on Snell’s law which states that excitation light is totally internally reflected at the interface between areas with different refractive indices, when the angle of incidence exceeds the critical angle. In a biological context, the media with different refractive indices are the cells (*n* ≈ 1.37) in aqueous solution plated on a coverslip (*n* ≈ 1.8). Upon total reflection, the photons slightly penetrate the aqueous medium thereby generating an electromagnetic field, which propagates parallel to the interface. This so-called evanescent wave exponentially decays with distance from the interface, therefore only fluorescent molecules close to the interface are efficiently excited. Depending on the angle of incidence, the excitation wavelength and the numerical aperture of the objective, the thickness of the evanescent field is usually within 150–200 nm from the glass-liquid interface. Fluorescence excitation by this evanescent wave results in images with very low background fluorescence (more than 2000-fold lower than in images collected by normal epifluorescence microscopy). This results in a high signal to-noise ratio, because virtually no out-of-focus fluorescence is collected.

**Figure 2:**
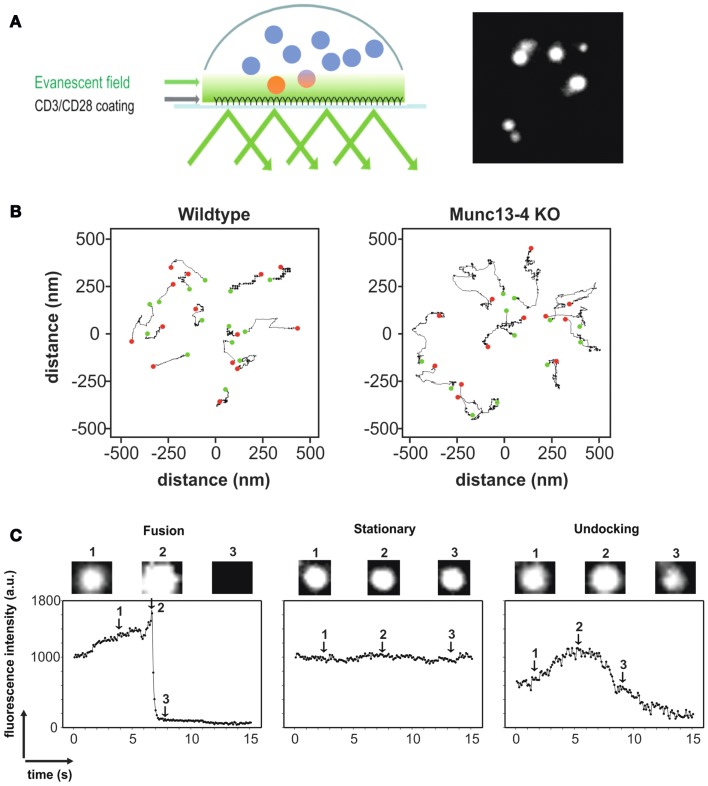
**(A)** Left, cartoon depicting the methodology of TIRFM in CTLs using anti-CD3/anti-CD28 antibody-coated coverslips to mimic an IS. Only LGs that are within the evanescent field (close to the IS; see Text for details) are fluorescent. Right, TIRFM image of CTLs transfected with granzyme B-TFP to specifically label LGs. **(B)** Representative trajectories of vesicles from WT (left) and Munc13-4 KO (right) CTLs to display their mobility. The green dot represents the starting position of the LG and the red dot represents the end position of the LG. The absence of the priming factor Munc13-4 leads to a significant increase in vesicle mobility in TIRFM (28). **(C)** Fluorescence profile of a fusing LG (left), a stationary LG (middle), and an LG that is undocking (right) as seen in TIRFM. Acquisition frequency was 10 Hz. Granzyme B-TFP was used to label LGs.

For this reason, TIRFM has become increasingly popular for the observation of LG exocytosis at the IS in CTLs and NK cells. An IS suitable for TIRFM has been induced in these cells in a variety of ways. One method consists of using planar lipid bilayers that carry ligands for activating specific receptors on the membrane of CTL or NK cells (24). Another method uses antibody-coated glass coverslips [Figure 2A; (25, 26)]. Imaging of CTLs transfected with F-actin that settled on antibody-coated glass coverslips showed the clustering and spreading of F-actin at the periphery of the cell, a typical characteristic of an early IS. Both methods successfully induce formation of synapses ideally suited for TIRFM imaging. Though LAMP1 is widely used as an endogenous marker for lytic granules, it also labels lysosomes. The debate on the maturation of lytic granules and their distinction from lysosomes is still ongoing (see above). Thus the use of markers specific for lytic granules such as perforin and granzyme B for studying their behavior and kinetics at the IS is preferable. Perforin goes through several stages of processing before becoming fully mature (see above). Therefore generating recombinant perforin appeared challenging. After perforin is delivered to LGs, its last 20 amino acids are cleaved by Cathepsin-L to generate fully functional perforin (27). This was demonstrated by the use of Cathepsin inhibitors to significantly decrease target-cell killing. Interestingly, target-cell killing by CTLs and NK cells from Cathepsin-L deficient mice is not diminished despite a reduction in the amount of processed perforin in the killer cells. This could indicate that other Cathepsins can compensate for the absence of Cathepsin-L. While Cathepsin-L is active only in the acidic pH of the granules, other cathepsins can function at a neutral pH and might also cleave perforin at the C-terminus after release from the LG. Human perforin from CTLs was indeed successfully cloned as a fusion construct with mCherry at the C-terminus (25). While it remains possible that the mCherry could indeed hinder Cathepsin-L from cleaving the last 20 amino acids at the C-terminus of perforin, this will not affect the validity of using perforin-mCherry as an LG marker. Granzyme B or FasL tagged with various fluorophores such as RFP, mTFP, mCherry, and pHluorin have been used as specific markers for LG studies using TIRFM. Co-transfection of perforin-mCherry and granzyme B-mTFP in human CTLs showed complete colocalization as expected and co-secretion at the TIRF plane mimicking an IS (data not shown). Our recent work in CTLs isolated from a Synaptobrevin2-mRFP knock in mouse demonstrated that Synaptobrevin2 mediates the final fusion step of lytic granules in CTLs (19). Synaptobrevin2 perfectly colocalized with mouse granzyme B and both were present at fusing LGs at the IS, thus providing an endogenous marker for lytic granules in mouse CTLs (see Figure 1C).

An additional advantage of TIRFM is that one can carefully distinguish and quantify pre-fusion events such as docking and priming. Docking is defined as the tethering of vesicles to the plasma membrane. The time that a vesicle resides in the TIRFM plane, referred to as the dwell time, is therefore a good measure of vesicle docking. The axial mobility of vesicles has also been used to quantify docking (29). Vesicle docking is followed by priming, which is defined molecularly as the formation of the trimeric SNARE complex, resulting in restricted mobility of the vesicle. Analyzing the mobility of vesicles in TIRFM can be used to characterize and differentiate the molecular states of priming and docking (30). An example of the quantification of the mobility of LGs from CTLs of wildtype and Munc13-4 knockout mice is shown in Figure 2B. Munc13-4 is considered to be a priming factor for LGs. A clear difference in the mobility of the vesicles could be seen, thereby establishing the correlation between less mobility and the state of priming. Analyses of the mean square displacement (MSD) over the increment of time or analyzing the caging diameter (CD) are well-established methods for analyzing the mobility of vesicles. However, the CD analysis is preferred since it also allows the quantification of the dynamic changes in the mobility itself, over time.

The fusion of lytic granules can be visualized in real time and quantified by TIRFM. Fast acquisition allows the analysis of the fluorescence intensity of a vesicle over time, to clearly distinguish between a vesicle that undergoes fusion, a stationary vesicle and a vesicle that is moving away from the plasma membrane (undocking, Figure 2C). In addition, the modes of fusion such as complete and incomplete fusion can also be analyzed by visualizing fusion pore opening (31). Their work implicated the existence of both fusion modes for LGs in primary NK cells. Incomplete fusion, also called “kiss and run,” also occurs in neuroendocrine cells and in several neuronal synapses as studied by imaging and combined patch-clamp and amperometric recordings (32).

Total internal reflection fluorescence microscopy has therefore been invaluable not only to specify the role of key players in LG release, but also to define the sequence of events that lead up to the final lethal hit. Actin, which is considered a marker for the early IS, was recently shown by TIRFM to also play a role in LG degranulation itself (33).

## Examining Lytic Granule Fusion by Combined TIRFM and Patch-Clamp Measurements

The final step of LG maturation is the fusion of LGs with the plasma membrane at the IS. As discussed above TIRFM is the method of choice to visualize LG dynamics at the IS as it enables the quantitative analysis of membrane-bound pre-fusion events like docking and priming. Furthermore it allows the real-time visualization and quantification of fusion by studying the kinetics of the loss of the fluorophore. However, TIRFM does not allow an estimate of the size of a docked, primed, or fusing LGs due to resolution limits defined by Abbe’s law (34). Immunogold labeling with perforin- and/or granzyme B-antibodies in human and mouse CTLs revealed ∼25 granules/cell with an average diameter of 700 nm, but yielded only static snapshots with unknown physiological relevance (15). Therefore, a method, which measures the size of a fusing LG, would be ideal.

The lipid bilayer of biological membranes separates the intracellular space from the extracellular solution surrounding the cell and acts as an electrical insulator between two conducting mediums. From a physical standpoint the cell membrane thus acts as a capacitor, and its specific capacitance has been calculated as 1 μF/cm^2^ (35). The membrane capacitance is proportional to the surface area of the cell. If an intracellular vesicle fuses with the plasma membrane, it increases the membrane capacitance proportional to the surface area of the vesicle. Conversely, if endocytosis occurs, the membrane capacitance decreases. Membrane capacitance measurements, as a highly quantitative readout for exocytosis of chromaffin granules, was introduced 30 years ago (36). Fusion of an individual chromaffin granule from mouse results in a capacitance increase of 1.3 fF (37) which translates to a diameter of about 200 nm. This value is in excellent agreement with morphological EM data of chromaffin granules (38).

**Figure 3:**
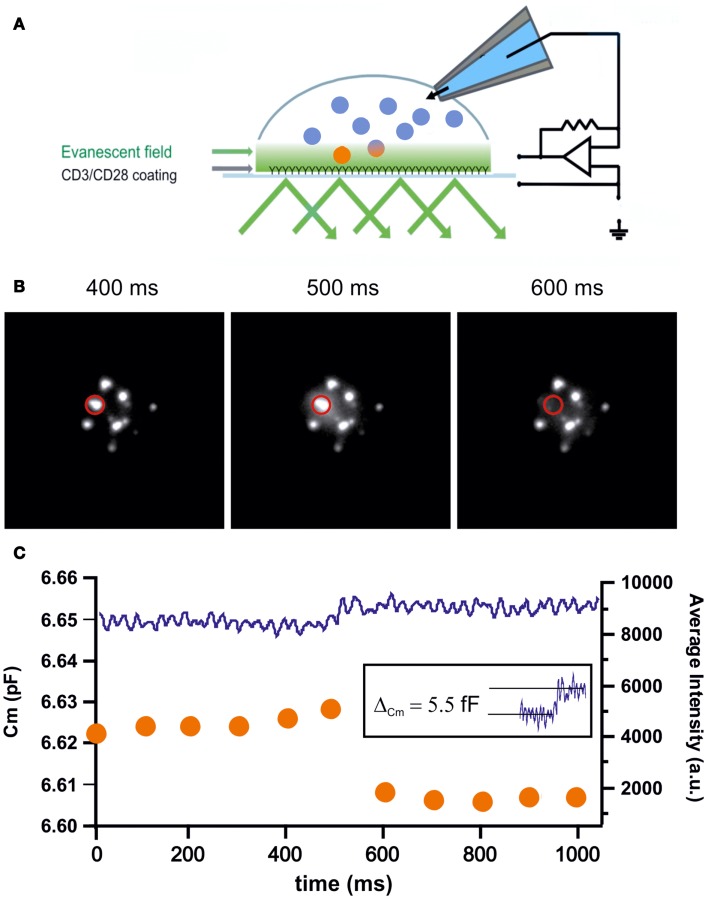
**(A)** Cartoon depicting the methodology of combined TIRFM/patch-clamp experiments. The coverslip was coated with anti-CD3/anti-CD28 antibodies to mimic an IS. CTLs were transfected with granzyme B-TFP to visualize LGs in TIRFM. Simultaneously, CTLs were patched with a pipet containing iso-osmolar solution containing 2 μM Ca^2+^ to trigger secretion. Capacitance measurements were performed using the Lindau–Neher technique implemented as the “sine + dc” mode of the software lock-in extension of the PULSE software (41). **(B)** Exemplary TIRFM images showing the fusion of an LG within one frame. The middle panel shows the typical “halo” that originates from dequenching and diffusion of the fluorophore. **(C)** Graph showing the simultaneous measurement of membrane capacitance (left axis) and fluorescence intensity (right axis) of the LG shown in **(B)**. Upon fusion the fluorescence steeply declines within one frame while in parallel the capacitance increases step-like. The capacitance increase of the fusing vesicle (5.5 fF; inset) corresponds to an LG diameter of 418 nm.

The measurement of membrane capacitance increases upon LG fusion from CTLs is complicated for two reasons. First, stimulated CTLs are highly mobile cells that tend to move through culture dishes while searching for potential target cells. This mobility makes patch-clamp recordings with a glass electrode technically quite challenging. Second, CTLs continuously add and retrieve proteins to and from the plasma membrane through exo- and endocytosis, mostly through recycling endosomes. For example, upon target-cell recognition TCR are integrated into the plasma membrane to form the cSMAC long before LGs arrive and fuse at the IS (39). Therefore, in order to distinguish between the fusion of LGs and other organelles, patch-clamp membrane capacitance measurements must be combined with TIRFM in order to visually identify LG fusion events that occur simultaneously with step-like capacitance increases [Figure 3A; (40)]. An exemplary trace of an LG fusion event is shown in Figures 3B,C. A sudden drop in fluorescence (orange trace) is accompanied by an increase in membrane capacitance (blue trace). The measured capacitance step of 5.5 fF relates to an LG diameter of 418 nm, considerably smaller than the estimate from EM data (15).

Besides the accurate determination of the diameter of fusogenic LGs, combined TIRFM/patch-clamp measurements enables the researcher to address other, important features of LG release. The unrestricted access to the interior of the cell through the patch pipette allows, for example, the manipulation of the intracellular calcium concentration to investigate whether the LG fusion process itself is calcium-dependent and, if yes, quantify the kinetics of this coupling.

## Outlook-Future Direction

We have described three high-resolution methods that enable the quantitative analysis of CTL function. Besides these techniques, novel microscopy techniques such as structured illumination microscopy [SIM (42)], stimulated emission depletion [STED (43)] and direct stochastic optical reconstruction microscopy [dSTORM (44)] have recently become available. The combination of these techniques will allow the investigation of CTL and NK cell function with unparalleled detail and resolution, promising a deeper understanding of this fundamental, immunological process.

## Conflict of Interest Statement

The authors declare that the research was conducted in the absence of any commercial or financial relationships that could be construed as a potential conflict of interest.
